# Safety Profile of PrePex Male Circumcision Device and Client Satisfaction With Adolescent Males Aged 13–17 Years in Zimbabwe

**DOI:** 10.1097/QAI.0000000000000799

**Published:** 2016-05-24

**Authors:** Mafuta Tshimanga, Karin Hatzold, Owen Mugurungi, Tonderayi Mangwiro, Getrude Ncube, Sinokuthemba Xaba, Pesanai Chatikobo, Patricia Gundidza, Christopher Samkange, Roy Dhlamini, Munyaradzi Murwira, Gerald Gwinji

**Affiliations:** * ZICHIRE, Harare, Zimbabwe;; † Population Services International, Harare, Zimbabwe;; ‡ Ministry of Health & Child Care AIDS and TB Unit, Harare, Zimbabwe;; § University Zimbabwe College of Health Sciences, Harare Zimbabwe; and; ‖ Zimbabwe National Family Planning Council, Harare, Zimbabwe.

**Keywords:** safety, PrePex, device, Adolescence

## Abstract

**Background:**

The safety and efficacy of the PrePex device for voluntary medical male circumcision (VMMC) has been demonstrated in studies in Rwanda, Uganda, and Zimbabwe, leading to the conditional prequalification of the device for use in adults. Because the majority of VMMC clients in the 14 priority countries are adolescents under 18 years, research to establish the safety and efficacy of the device for males <18 years is required.

**Methods:**

One-arm, prospective study included 402 adolescents, aged 13–17 years, using PrePex device between August 2013 and January 2014 at a VMMC centre in Harare. Endpoints are number and grade of adverse events associated with device circumcision, time to complete wound healing, client satisfaction with the procedure, and outcome.

**Results:**

The rate of medical ineligibility among adolescent males was high; 237/402 (35.9%) of study participants had to be excluded based on medical reasons. The severe/moderate adverse event rate was low at 2/402 (0.5%). No device displacements/self-removals were observed. Time to complete wound healing was shorter than in adults; 367/398 (92.2%) adolescents had completed wound healing by day 35, whereas 90% of adults had completed wound healing by day 56 as demonstrated in previous studies. Overall, adolescents were highly satisfied with the results of their circumcision.

**Conclusions:**

The study demonstrates that the PrePex device can be safely used in adolescents aged 13–17 years. The significant proportion of males opting for surgical circumcision and the high medical ineligibility suggest that surgical circumcision needs to be provided alongside PrePex services in programs targeting young age groups.

## BACKGROUND

Voluntary medical male circumcision (VMMC) reduces the risk of heterosexually acquired HIV infection in men by 60%^1–3^ and represents one of the most cost-effective HIV prevention interventions that exist today.^4^ Modeling studies conducted in 2009 and 2011 estimated that circumcising 20.3 million men aged 15–49 years in 14 priority countries in eastern and southern Africa within 5 years, and sustaining 80% coverage thereafter, could avert 3.4 million HIV infections within 15 years and save $16.5 billion in treatment costs.^5,6^ Following World Health Organization (WHO) guidance in 2007, VMMC programs have been launched in 14 priority countries in sub-Saharan Africa as a part of a comprehensive HIV prevention strategy. Projections show that a cumulative total of 10 million men would have been circumcised by the end of 2015 since the first national VMMC programs started in 2007.^7^ Devices have the potential to accelerate VMMC service delivery by making the procedure quicker, easier to perform and scalable, more acceptable, and potentially more cost-effective.^8,9^ One promising device for adult VMMC is the PrePex device. Following results from studies in Rwanda,^10–12^ additional research with the device was conducted in Zimbabwe to establish its safety, efficacy, and acceptability among adults.^13–15^ The data from 3 consecutive studies were submitted to WHO, which led to the prequalification of the PrePex device in May 2013 for use in adults.^16^ The WHO *Framework for Clinical Evaluation of Devices for Male Circumcision* also recommends that bridging studies are conducted to assess device safety in a wider range of clients than those included in the prequalification trials and studies, where inclusion and exclusion criteria may have restricted participation to males over a certain minimum age.^17^ These studies are required to investigate the safety and effectiveness of extending the use of PrePex to age groups younger than those included in the prequalification studies. In particular, the safety and acceptability of PrePex is not known in clients <18 years of age, who, it was speculated, might be more likely than adults to dislodge the device through manipulation or masturbation. In addition, since a large proportion of VMMC clients in the 14 priority countries have been adolescents aged <18 years, research to establish the safety and efficacy of the device when used in males under 18 years is urgently needed, if the device should be integrated with routine VMMC programming.^18^ A bridging study was conducted to assess the safety and acceptability among a total of 400 male adolescents, aged between 13 and 14 years and 15–17 years. Before the PrePex adolescents bridging study, a device sizing study was conducted with 199 adolescents to determine the additional smaller sizes of the device that would be required to circumcise adolescents aged 13–17 years. The results from the sizing study provided information for the manufacturer to produce 5 additional smaller sizes to fit some of the younger adolescent males. Safety results from 402 adolescent participants recruited in the study between August 8, 2013 and January 23, 2014 are presented here.

## METHODS

### Design

The PrePex adolescents bridging study was a one-arm, open-label prospective study. All circumcisions were conducted at the Zimbabwe National Family Planning Council Spilhaus PrePex study site in Harare, where conventional surgical VMMC services were also provided. The study consisted of 2 phases: phase I included 199 (49.5%) adolescents in the age group 15–17 years; and phase II included 203 (50.5%) adolescents in the age group 13–14 years, giving a total of 402 subjects. Initially, 50 study participants (aged 15–17 years) were circumcised with the PrePex device by physicians, and the safety of the device was assessed. Once safety of the device had been established based on the first 50 cases, procedures were conducted by nurse providers for the remaining 352 participants under the supervision of study physicians. All review days were in reference to post placement days with the day of placement being day 0. Follow-up of study participants was conducted on day 0 immediately after the procedure to confirm proper placement, on day 2 for participant assessment with the device in situ, on day 7 for device and foreskin removal, on day 9 for review post removal, and thereafter weekly up to 56 days postplacement or until epithelialization of the wound was achieved.

### Study Objectives

The primary objective of this study was to assess the safety of PrePex procedures when used with adolescent males aged 13–17 years. Successful circumcision was defined as removal of sufficient foreskin such that the coronal sulcus was visible with the penis in a flaccid state. All device circumcision related adverse events (AEs) were classified using the consensus Population Services International/WHO/College of Surgeons of East, Central, and Southern Africa Adverse Event Action Guide^19^ and specific device related AE guidelines that had been used by WHO for the review of clinical data on device safety by the WHO Technical Advisory Group on Innovations in Male Circumcision^16^ AE definitions stipulated specific, distinct designations for circumcision failure, device displacements, self-removals, and device malfunctions. Participants were assessed for AEs at every follow-up visit for type, severity, and treatment of the event. AEs related to pain were measured using a visual analogue score (VAS) with possible values of 0, 2, 4, 6, 8, and 10 where 0 corresponded to “no pain at all” and 10, to “worst pain imaginable.”^20^ The pain assessments were made at 15 minutes post placement, 1–3 hours post placement, preremoval, during foreskin removal, during the device removal, 1 minute post removal, and at every subsequent weekly follow-up visit. Secondary objectives of the study were to determine the time to complete healing after PrePex circumcision, defined as complete wound epithelialization without any scab, and to evaluate the acceptability of PrePex procedures among clients and providers.

Three in-depth interviews were scheduled for 50% of the 402 study participants (n = 201). Data were collected at 3 time points: preplacement (immediately after clinical assessment); at 14, and at 60 days post device application. Adolescents were asked to rate the effects of the PrePex circumcision on activities of daily living, including its effect on school attendance, on a 5-point scale with values ranging between “strongly disagree” and “strongly agree.”

### Study Site and Recruitment

The safety study was implemented at Zimbabwe National Family Planning Council Spilhaus VMMC centre in Harare. Males aged 13–17 years, who voluntarily presented and subsequently attended the VMMC group education session, were offered either (1) routine (nonresearch) conventional surgery or (2) PrePex device circumcision, as part of the study. Those who decided for surgery were not included in the study. Those who chose device circumcision were asked to have their guardian sign the informed consent form and to sign the assent form before further counseling and assessment for eligibility was conducted. Potential participants who did not meet the inclusion criteria or who opted for routine surgery were offered conventional surgical circumcision. The number and reasons for such exclusions were recorded.

### Eligibility

**FIGURE 2.**
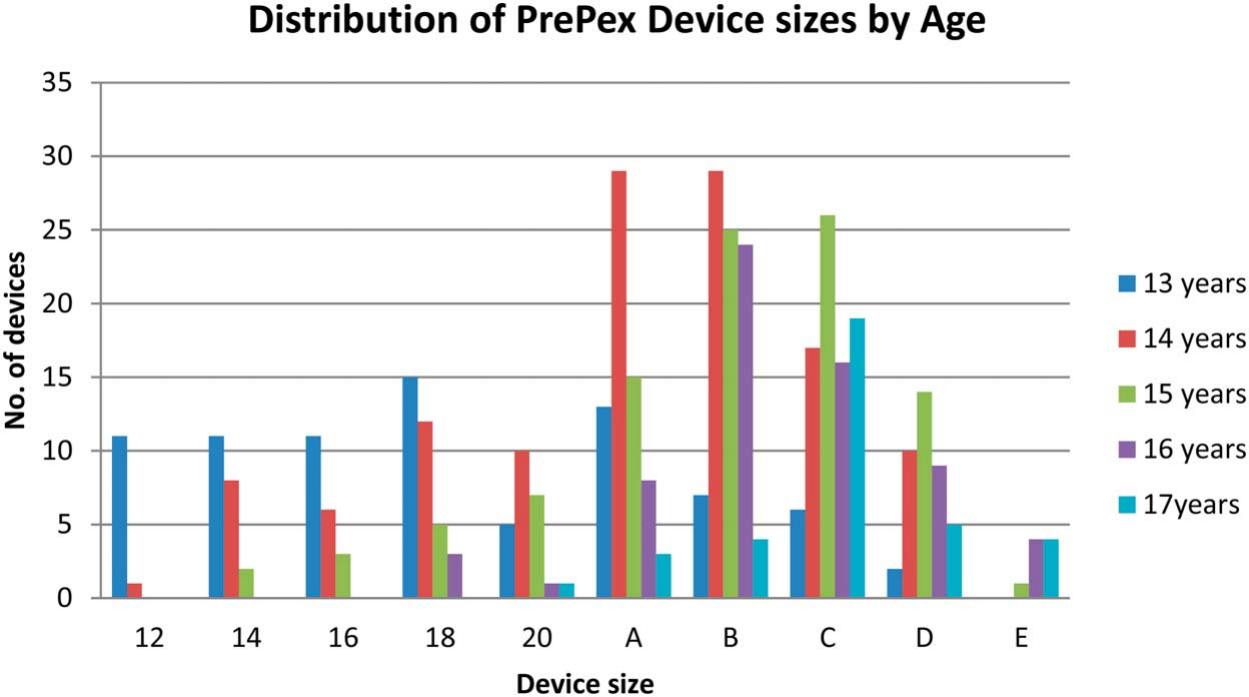
Distribution of PrePex device size by age, N = 402. Sizes: A: N = 68 (16.9%), B: N = 89 (22.1%), C: N = 84 (20.9%), D: N = 40 (10%), E: N = 9 (2.2%), size 12: N = 12 (3%), size 14: N = 21 (5.2%), size 16: N = 20 (5%), size 18: N = 35 (8.7%), and size 20: N = 24 (6%).

Inclusion criteria were: age 13–17 years, HIV-uninfected, good general health and clinically free of sexually transmitted infections, consent by parent and assent by participant, provide contact information, and able to present for follow-up services as per study protocol. A participant was excluded from the study if his penis did not fit any of the 10 PrePex sizes, or if he had a medical contraindication to male circumcision or participating in the study. The PrePex device size distribution is presented in Figure 2. Sizes A-E were previously available for circumcisions in males >18 years of age and sizes 12–20 were specifically created for the males in this study who needed smaller sizes.

### Data Analysis

Data from 402 study participants were entered into a database in Service Provisioning System Software and analyzed to ascertain the proportion ineligible and to document reasons for exclusion, the percentage of PrePex clients failing to return to clinic at scheduled review dates, and the percentage of AEs including device displacement/detachment/self-removal/device malfunction. The proportion of men with complete wound healing at specific review dates was calculated. Acceptability features, time to return to normal activity, and satisfaction with the postcircumcision cosmetic results were summarized.

### Ethical Conduct

The Medical Research Council of Zimbabwe approved this study. The study was monitored by international monitors (Research Support Centre, College of Medicine of Malawi) for Good Clinical Practices and international standards such as ISO 14155. In addition, a Data Safety Monitoring Board was established to provide required oversight in all phases of the trial.

## RESULTS

### Study Population and Eligibility

**FIGURE 1.**
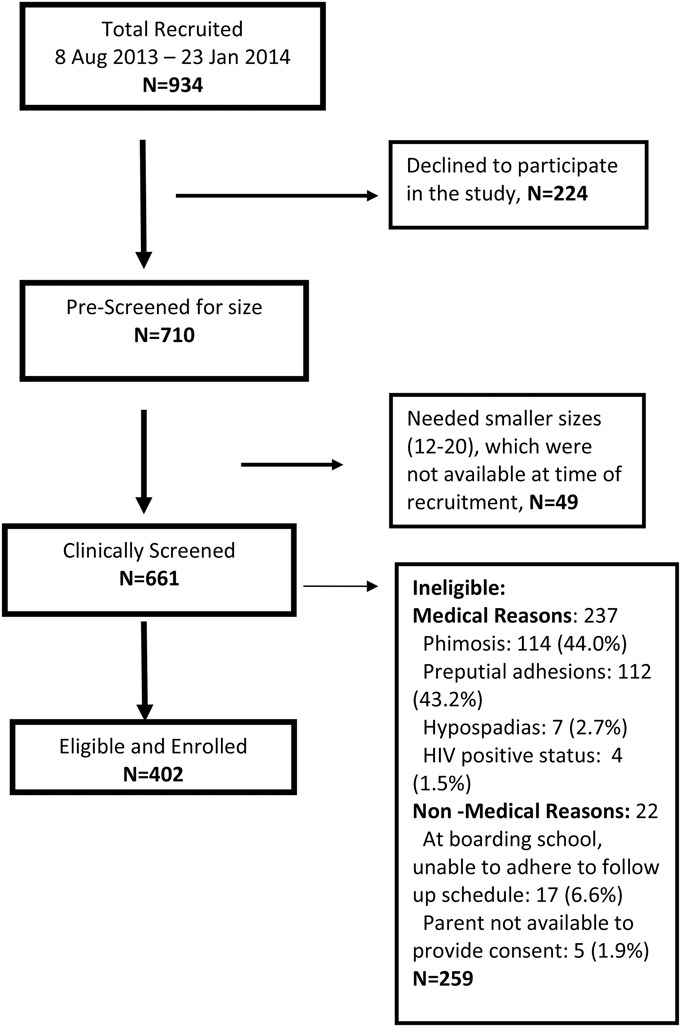
Flow chart study participants enrolment/eligibility.

A total of 934 clients from the eligible age group of 13–17 year old males presented at the Harare Spilhaus VMMC site between August 8, 2013 and January 23, 2014. Of the 934 offered PrePex, 224 declined and were offered conventional surgical VMMC. Seven hundred ten clients consented, assented, and prescreened for device sizing. Of those, 49 were excluded because the required smaller device sizes were not available at the time of recruitment, due to supply constraints (when the study started, the specific smaller sizes, size 12–20, had not yet been received) and 661 were further clinically screened for PrePex eligibility (Fig. 1).

### Eligibility

Of the 661 participants who were further clinically screened for eligibility, 259 (39.2%) were excluded for medical or nonmedical reasons (Table 1). None of them were excluded because they needed a device size smaller or larger than the 10 available sizes.

**TABLE 1.**
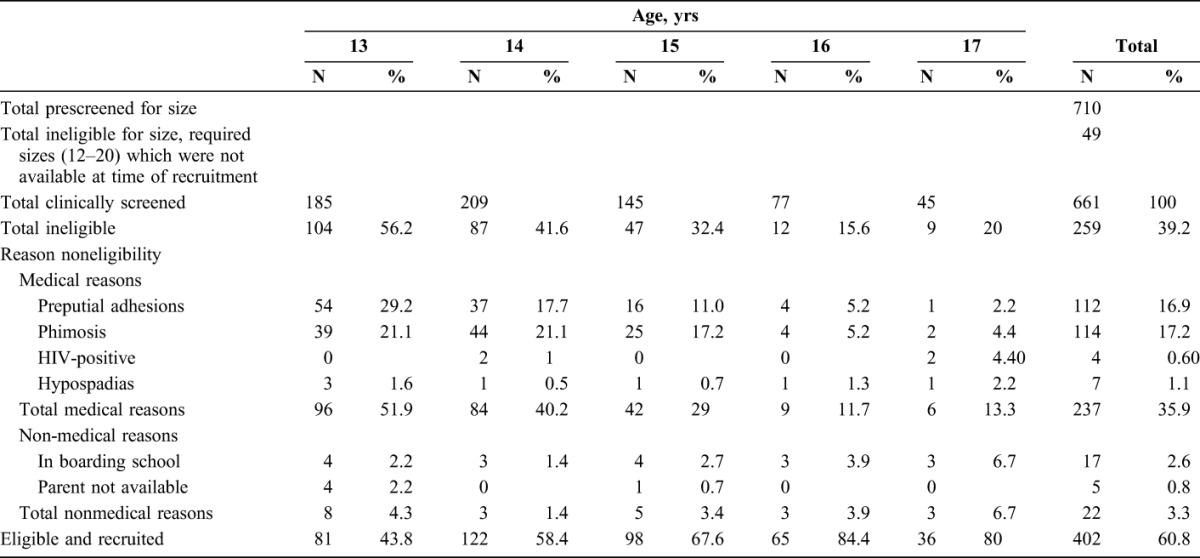
PrePex Male Circumcision Eligibility Among Participants by Age and Reason for Ineligibility

#### Medical Reasons

The majority of adolescents (N = 237, 35.9%) were excluded for medical reasons. The most common medical reasons for ineligibility were phimosis (17.1%), preputial adhesions (16.9%), hypospadias (1.1%), and HIV-positive status (0.6%). All HIV-positive participants were counseled with their guardians and referred to the national antiretroviral therapy program for medical treatment and further investigations (Table 1).

#### Nonmedical Reasons

Nonmedical reasons for ineligibility included 22 (3.1%) participants who were either attending boarding school and could not adhere to the review dates, or whose guardians were not available to provide consent and were therefore not eligible to participate in the study (Table 1).

#### Device Size

Figure 2 shows the distribution of the device sizes by age. Ten different device sizes were successfully placed with the 402 participants in the adolescents PrePex safety study. The most common device sizes used were size B (22.1%) and size C (20.9%). The least used sizes were size E (2.2%) and size 12 (3%). One hundred twelve (27.9%) study participants required smaller size devices (size 12–20).

#### Adverse Events

There were no device displacement and no self-removals recorded. Mild AEs included mild localized oedema post device removal. One moderate and 1 severe adverse event occurred in 2 study participants. The moderate AE occurred in a 13-year-old study participant with penis swelling, mild blistering of the shaft skin distal from the device and, dry foreskin covering the meatus on day 5 resulting in voiding problems. The device and dry foreskin were removed on day 5, and the wound healed without complication. The severe AE occurred in a 13-year-old study participant, who had insufficient foreskin removal. This case was classified as a severe AE because management included a surgical procedure. The device was removed on day 7 and the client healed completely on day 35, but had phimosis. The client was operated on day 90 with dorsal slit method.

### Pain Assessment

The VAS pain level reported by all participants was summarized for each step of the procedure as shown in Table 2. None of the pain scores measured at any time with any of the participants was classified as moderate or severe. Highest pain scores were recorded at device removal with 307/402 participants reporting a VAS pain score of 2 and 69/402 reporting a VAS pain score of 4.

**TABLE 2.**
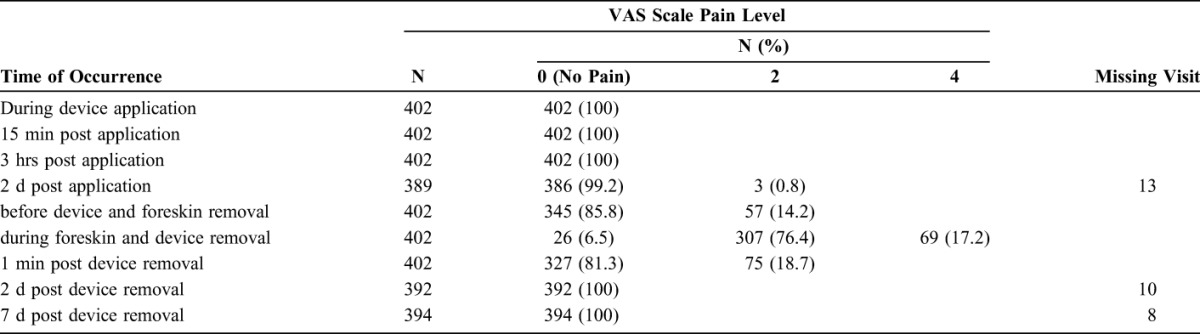
Summary of VAS Pain Level in the 402 Participants at Key Time Points

### Time to Complete Healing

Time to complete healing was assessed for 398/402 participants who attended review visits at the scheduled time. Four/402 (1.1%) participants did not return for follow-up visits at different time intervals (2 on day 42, 1 on day 35 and 1 on day 28) and healing time could not be determined for these. When contacted by telephone on the day of the scheduled visit, all 4 participants reported that the wound had healed. Among the 398 clinically assessed for healing, 367 (92.2%), 394 (99.9%), and 398 (100%) were deemed fully healed by days 35, 42, and 63, respectively.

### Acceptability and Satisfaction Parameters

When asked about their satisfaction with the circumcision, 96.9% and 96.1% of interviewed study participants reported being “very”/“extremely” satisfied during interviews at day 14 and 60 respectively. Whereas reports of odor were not solicited, none of the participants complained of odor.

On day 14, 11.8%, 16.5%, and 25.3% strongly or somewhat agreed that the circumcision affected their ability to sit, to walk, and their ability to sleep, respectively. Of the participants, 17%, 25.2%, and 34.2% strongly or somewhat agreed that the circumcision affected their ability to do housework, their school attendance, and their ability to participate in sports, respectively (Table 3).

**TABLE 3.**
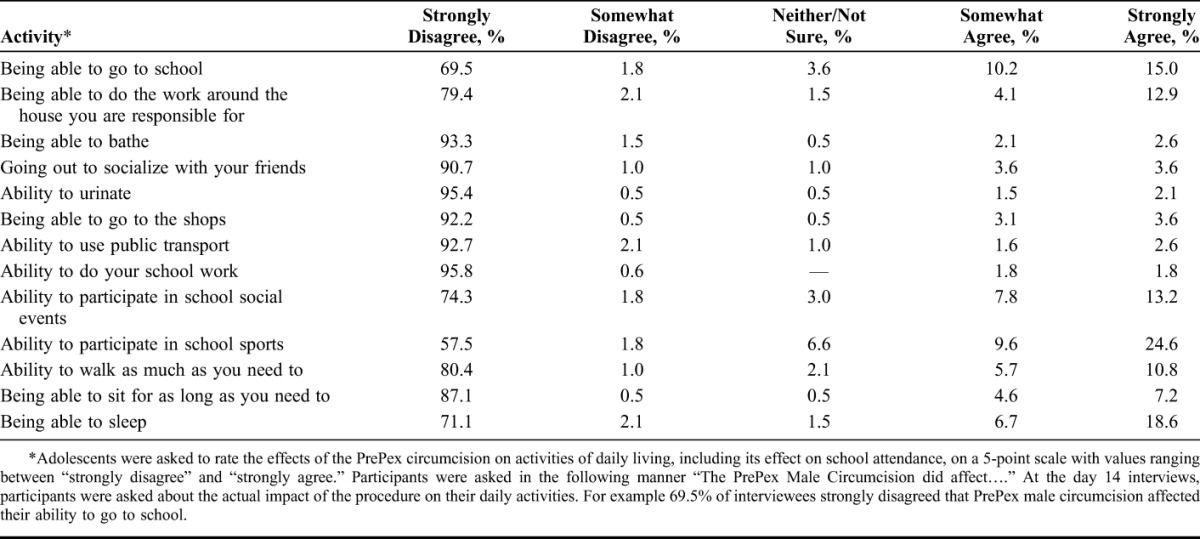
Impact of PrePex Male Circumcision on Daily Activities, Interviews Day 14 Post Device Application, N = 195

## DISCUSSION

This study demonstrates that the PrePex device can be used safely in adolescents when the procedure is performed by physicians and registered general nurses. The rate for moderate and severe AEs of 0.5% was lower than what had been reported in previous studies with adults. Furthermore, there were no device displacements or self-removals. In previous adult PrePex studies from Zimbabwe, a moderate/severe AE rate of between 0.3% and 0.7% had been reported, including severe AEs because of device displacement and self-removal.^13–15^ The WHO Technical Advisory Group reported an AE rate from 8 studies in males >18 years of age with 2417 PrePex device placements of 1.7%.^16^ A recent study in Uganda reported AEs in 1.6% of adult participants,^21^ and in another study from Kenya an AE rate of 5.9% was reported.^22^

The relatively lower moderate and severe AE rate without any cases of device displacements/self-removals might be explained by the fact that most adolescent participants were not yet sexually active. This may have reduced their risk of device displacement and self-removal, while engaging in sexual activity while wearing the device. The low AE rate satisfies the concerns of experts who feared that younger clients may be more likely to dislodge the device through manipulation or masturbation.

The VAS pain scores reported at various time points during application, while wearing the device and at removal in this study were generally lower than those observed among adult study participants in previous studies in Zimbabwe,^13–15^ using the same providers, the same pain score grading system, and pain relief measures for study participants. None of the study participants complained of VAS pain scores higher than 4, which relates to mild pain. As in other studies, no injectable anesthesia was used for pain relief at application or removal of the device.

Healing after PrePex male circumcision is by secondary intention, and studies among adults have shown that wound healing with PrePex takes about 7 days longer than with conventional surgery in adults, measuring from the time of device application, including the 1 week of device in situ. In the comparison study in Rwanda,^23^ the mean time to complete healing was 38 (SD = 12.1) days following PrePex placement compared with 23 (SD = 7.5) post surgery and the overall mean healing time among adults over 5 PrePex studies was recorded as 42.3 (SD = 7.8) days.^16^ In this study, a shorter mean healing time of 31.9 (SD = 5.47) days was observed. Data from PrePex pilot implementation studies in Mozambique, South Africa, and Zambia showed that 88.3%, 87.8%, and 38.7% of adult participants, respectively had complete wound healing on day 49.^24^ Furthermore, younger adolescents aged 13–14 years seemed to heal faster than older adolescents (15–17 years). In the absence of a comparison surgical arm, it is assumed that the relatively shorter healing time among adolescent PrePex clients is due to the faster wound healing process in younger clients versus a difference due to choice of method.

One of the most important and limiting factors for PrePex male circumcision in adolescents aged 13–17 years is the relatively high rate of medical ineligibility observed in this study, especially for the younger ages 13–14 years. Although the overall medical ineligibility was 39.2% among all age groups, an estimated 51.9% and 40.2% of the 13 and 14-year-old participants, respectively were ineligible for the PrePex procedure, mainly because of physiological immaturity with phimosis and preputial adhesions. Medical ineligibility decreased as the age of the participants increased, with 29.0%, 11.7%, and 13.3% of the 15, 16, and 17-year-olds, respectively, found ineligible for the PrePex procedure. This compares to a medical ineligibility rate of 5.9% among adult men with the major reasons being phimosis, narrow foreskin opening, and tight frenulum observed in 8 studies conducted in Rwanda, Uganda, and Kenya.^16^ The high rate of medical ineligibility has consequences on program planning and implementation if PrePex is introduced in national VMMC programs, as this limitation requires that surgical circumcision services be readily available if age groups <18 years are targeted. The cost implications of these exclusions need to be considered by decision makers, as do the proportions of potential participants who decline the use of PrePex in favor of surgical circumcision (24% in this study). The cost analysis of integrating the PrePex medical device into a VMMC Program in Zimbabwe, assuming a device unit cost of $20, found no evidence that introducing the PrePex device would result in increased efficiency of the VMMC program in terms of reducing the unit cost, when PrePex would be provided together with the surgical procedures at a mixed site.^25^

Overall, participants were very satisfied with the PrePex procedure. Pain management could be enhanced by specific messages about when pain is most likely to occur and by additional pain relief during device removal.

This study featured several strengths. The study was large enough to yield reasonably precise estimates of moderate/severe AE rates, and the multiple follow-up visits offered a precise estimate of the healing time. The weakness of the study was that it had no comparison surgical arm to compare AE rates and healing time. In addition, interpretation of complete healing may have been inherently subjective and may have varied between providers. This also applies to the measurement and classification of pain experienced by study participants during the procedure.

In conclusion, the PrePex device was an effective, safe, and acceptable method of male circumcision in adolescent males 13–17 years. The major drawback is the high medical ineligibility for device use in this age group, which requires availability of surgical male circumcision services.
